# Using quality improvement to standardise and enhance the use of the national early warning score (NEWS) in an old age psychiatry inpatient setting

**DOI:** 10.1192/bjo.2021.153

**Published:** 2021-06-18

**Authors:** Fraser MacNicoll, Mong Sun Tung, Brion McGowan

**Affiliations:** 1 Psychiatry of Old Age, NHS Lothian; 2 NHS Lothian

## Abstract

**Aims:**

Within an inpatient old-age psychiatry setting, there is an increased risk of acute physical deterioration secondary to age, comorbidities and reduced physiological reserve. Numerous recent clinical incidents highlighted late recognition of physical deterioration within this population. We assessed the use of the NEWS, a system for scoring physiological measurements, in an old-age psychiatry ward and subsequently attempted to improve performance of obtaining physical health observations in this cohort of patients.

**Method:**

Retrospective pre- and post- quality improvement study in a twenty bed Old Age Psychiatry Ward in East Lothian Community Hospital, Haddington, Scotland. Data were collected from 12th October – 16th November, 2020 (pre- period) and from 16th November 2020 to 15th February, 2021 (post- period). The primary process measure was ensuring all patients had at least one full set of physical observations at least once a week, or more frequent as deemed clinically appropriate. Secondary measures included ensuring NEWS scores were accurately calculated and improved documentation. This was tracked using a run chart. Improvement activities focused on increased awareness, effective training, key stakeholder buy-in and reviewing trust policy.

**Result:**

The percentage of NEWS documented for all patients at least once a week improved from a mean of 28.7% (31/108) in the 6 weeks prior to intervention, to a mean of 71.4% (125/175) in the following 13 weeks. The minimum required physical observations required to accurately calculate a NEWS improved from 51.6% (16/31) pre-intervention to 95.2% (119/125) post-intervention and NEWS being calculated correctly increased from 80.6% (25/31) to 96% (120/125). Documentation of a reason why physical observations were not taken increased from 2.5% (2/77) to 62% (31/50) pre- and post- intervention respectively.

**Conclusion:**

This quality improvement project highlighted that recording of physical observations and use of NEWS was inadequate in this setting, increasing the risk of a delay in identification of acute physical deterioration and thus increase morbidity and mortality. Introducing simple measures and standardising the NEWS assessment process, along with senior nursing and medical oversight, greatly enhanced acquiring and recording of physical observations and NEWS scores. This quality improvement project has shown that practical solutions and staff education can increase efficacy and are hoping further input can consolidate the gains achieved and lead to continued improvements.

